# Resilience, Perceived Stress, and Depressed Mood in Women Under in Vitro Fertilization Treatment

**DOI:** 10.1007/s43032-021-00685-1

**Published:** 2021-09-14

**Authors:** Carmen Fernandez-Ferrera, David Llaneza-Suarez, Daniel Fernandez-Garcia, Vanesa Castañon, Cristina Llaneza-Suarez, Placido Llaneza

**Affiliations:** 1 Universidad de Oviedo, Departamento de Cirugía y Especialidades Medico Quirurgicas, Hospital Universitario Central de Asturias, Av. Roma, s/n, 33011 Oviedo, Asturias Spain; 2 FIV4, C/ La Merced 22, 33201 Gijon, Spain; 3 Quirinal Primary Healthcare Centre, C/Fuero de Avilés, 18, 33401 Avilés, Asturias Spain; 4 Department of Obstetrics and Gynecology, Hospital Universitario Central de Asturias, Av. Roma, s/n, 33011 Oviedo, Asturias Spain; 5 Hospital Da Costa Burela, Rafael Vior s/n 27880 Burela, Lugo, Spain

**Keywords:** In vitro fertilization, Perceived stress, Resilience, Depressed mood

## Abstract

It has been suggested that women who display higher resilience levels may have less psychological distress during IVF. The aim of this study was to evaluate how infertile women deal with perceived stress, depressed mood, and sleep disturbances at the first IVF attempt and after one or more negative IVF outcomes depending on their level of resilience. An observational, cross-sectional study was carried out in a sample of 207 infertile women undergoing IVF procedures. The participants completed the short version of the Connor-Davidson Resilience Scale (CD-RISC), the short version of the European Spanish Version of Perceived Stress Scale (PSS-10), the Center of Epidemiologic Studies Depression Scale (CESD-10), and the Jenkins Sleep Scale (JSS). The relationship between CD-RISC scores ranked according to percentiles and mean PSS-10 scores revealed that women with strong resilience had lower perceived stress. After splitting the sample according to CD-RISC percentiles, differences were observed only at the first IVF attempt and the observed protective effect of high resilience scores appears to disappear following a negative IVF outcome. Women with high resilience are less likely to suffer from perceived stress or depressed mood during their first IVF attempt, this protective effect appears to be lost after a negative outcome.

## Introduction

Infertility, defined as failure to achieve pregnancy within 12 months of unprotected intercourse or therapeutic donor insemination in women younger than 35 years or within 6 months in women older than 35 years, affects up to 15% of couples in the USA [1]. The prevalence of infertility varies worldwide ranging from less than 5% to over 30% with increasing rates thought to be primarily due to lifestyle factors such as the current trend of postponing childbearing, smoking, environmental influences, sexually transmitted diseases, or obesity, with a large proportion of these couples seeking medical intervention to conceive, particularly by in vitro fertilization (IVF) [2–4]. A diagnosis of infertility and the need for IVF can have a negative impact on the relational, sexual, and psychosocial well-being of couples, while rates of stress, anxiety and depression are higher among IVF patients than the general population [5]. These negative effects of infertility are stronger for women than for male partners and could be associated with poor quality of life and with a high withdrawal rate in IVF treatments [6].

In psychology, the term resilience refers to a complex and dynamic multidimensional construct, which results from the interaction between neurobiological, social, and personal factors and indicates the ability to adaptively cope with stress and adversity to maintain a normal level of physical and psychological functioning. It has been suggested that women who display higher resilience levels may have less psychological distress during IVF, but it is not stated if low resilience status in women undergoing IVF treatments should be considered as a risk factor for lower quality of life, a high probability of withdrawal or long-term emotional effects [7–9].

It is thus plausible to think that women with high resilience levels are better able to cope with perceived stress, depressed mood, and sleep disturbances in comparison with less resilient women. It is unclear, however, as to whether this potential protective effect could persist throughout several IVF attempts. The aim of this study, therefore, was to evaluate how infertile women deal with perceived stress, depressed mood, and sleep disturbances at the first IVF attempt and after one or more negative IVF outcomes depending on their level of resilience.

## Material and Methods

### Design and Patient Population

An observational, cross-sectional study was carried out in the Hospital Universitario Central de Asturias (Oviedo, Spain). The sample was recruited between 1 January 2016 and 31 December 2018 from women referred for initial or repeated IVF treatment that met the following inclusion criteria: they were seeking IVF, were over 18 years old and younger than 40 years old, and were able to complete the scales and sign the written consent form. In order to avoid potential sources of bias, women with a previous diagnosis of a malignant tumor, chronic, or psychological disease, and those known to have a documented substance abuse or alcohol addiction were not eligible for recruitment.

### Clinical Protocols

The day before beginning COH, one of the researches collected data related to age, parity, current height and weight, cause of infertility, infertility length, and the number of cycles to be carried out and the participants completed the short version (10 items) of the Connor-Davidson Resilience Scale (CD-RISC), the short version (10 items) of the European Spanish Version of Perceived Stress Scale (PSS-10), the 10-item Center of Epidemiologic Studies Depression Scale (CESD-10), and the Jenkins Sleep Scale (JSS). The CD-RISC consists of 10 items, each of which can be scored from 0 (never) to 4 (always). The total score ranges from 0 to 40 points, with scores above the 75^th^ percentile taken to indicate high resilience, and those below the 25^th^ percentile indicating low resilience. The scale has been tested in several contexts and has also been validated in the Spanish population [10, 11]. The PSS-10 was designed to measure the degree to which individuals appraise situations in their lives as stressful and the European Spanish version has been validated for the Spanish population [12, 13] along with the CESD-10 Scale for evaluating depressed mood [14, 15] and the JSS for measuring sleep disturbance [16, 17].

COH was carried out with GnRH subcutaneous 150–300 IU recombinant FSH (Gonal-F; Serono Labs) or hMG (Menopur; Ferring Labs) used from day 2 of the menstrual cycle. On the 6th day of the stimulation, subcutaneous 0.25 mg/day Ganirelix (Orgalutran; MSD Labs) was administered according to a fixed protocol. For each cycle, two-dimensional transvaginal ultrasound (TVU) was performed on treatment days 1, 6, and 10, recording the number and size of follicles. Ovulation was triggered by subcutaneous 0.25 mg hCG (Ovitrelle; Serono Labs) when the diameter of one or more follicles was over 17 mm, and the follicles were punctured 36 h later. Embryos were assessed daily according to ESHRE criteria until the time of embryo transfer, which was day 3 or 5 after fertilization. The number of transferred embryos was one or two. Pregnancy was diagnosed with the use of serum quantitative β-hCG and 3 weeks later by TVU for verification of embryonic cardiac activity. Pregnancy monitoring and deliveries were carried out in our hospital or other associated institutions. All the women were followed until they had a live birth, decided to withdraw from IVF treatment, or completed three failed cycles in cases of negative outcomes, but they only completed the scales in one cycle

### Statistics

A sample size of over 201 women was considered necessary to provide accuracy of 6% with a confidence interval of 95% considering that 25% of the women presented a high value of resilience and another 25% a low value [Sample size: (1.96)^2^ × 0.25 × 0.75 / (0.06)^2^ = 201 women].

A descriptive analysis of the data was conducted to obtain frequency distributions. For qualitative variables the mean and median were used as measures of central tendency, while measurements of dispersion were provided by the standard deviation, interquartile range and percentiles (25^th^–75^th^). The relationship between qualitative variables was analyzed using the Pearson Chi-squared test. Group comparisons of quantitative variables were conducted using Student’s *t* test for independent samples or Mann-Whitney test in the case of two groups, while ANOVA or Kruskal-Wallis test was used for three-group comparisons along with Tukey’s and Nemenyi test.

Statistical analyses were conducted using the R (R Development Core Team) program, version 3.3.1. [R: A language and environment for statistical computing. Vienna, Austria. Available at http: //www.r-project.org/ (ISBN 3-900051-07-0)].

### Ethical Considerations

The research protocol of the study was reviewed and approved by the Asturias Ethical Committee, Oviedo, Spain. All women were informed about the research (purposes, tools used and clinical protocols), and written consent was obtained from each participant.

## Results

A total of 265 infertile women undergoing IVF procedures with controlled ovarian hyperstimulation (COH) in an antagonist protocol were offered the opportunity to participate in the study. Fifty-three women declined the invitation to participate, did not adequately complete the scales, or voluntarily withdrew after providing written consent, while five women did not complete the study because of treatment cancellation due to poor ovarian response. Therefore, the final sample size for analysis was 207.

All women were in a heterosexual relationship, and male factor infertility was the only causal or contributing factor involved in the inability to conceive in 34.6% of the couples. Two women had previously had a live birth without IVF with a different partner and 49 women had previously been pregnant, but without a resulting live birth. One hundred and five women (50.7%) were undergoing their first IVF attempt, 71 women (34.4%) their second IVF attempt, and 31 (15%) their third IVF attempt. Characteristics and possible cofounders that could contribute to the overall interpretation of the results according to the number of IVF attempts are shown in Table 1.

**Table 1:** General characteristics of the participants according to IVF attempt

	1^st^ IVF (*n* = 105)	2^nd^ IVF (*n* = 71)	3^rd^ IVF (*n* = 57)	*p* value
Age,(years)	37.0 [3.0]	36.0 [4]	37.0 [4]	n.s.
Body mass index, kg/m^2^	23.0 [5.0]	24 [4.0]	24.0 [7.0]	n.s.
Waist circumference, cm	82.24 (18.86)	79.41 (7.88)	81.92 (11.26)	n.s.
Smoking habit	17.1%	23.9%	12.9%	n.s.
Years attempting to get pregnant	3.38 (1.97)	2.97 (1.35)	3.66 (2.61)	n.s.
History				
Previous miscarriage	16.2%	32.4%	29.0%	n.s.
Previous live birth	1.0%	1.4%	0%	
Nulligravida	82.9%	66.2%	71.0%	n.s.
Cause of infertility				n.s.
Female	17.1%	22.5%	19.4%	
Male	34.3%	35.2%	32.3%	
Multiple factors	24.8%	29.6%	22.6%	
Unexplained	23.8%	12.7%	25.8%	
Live birth after current treatment *(n*)	18.1% (19)	21.4% (15)	6.5% (2)	

Data are presented as %, mean (standard deviation) and median [interquartile range]. Chi square a *T* test were employed

The general characteristics of the women and their CD-RISC scores, ranked according to their position above the 75^th^ percentile and below the 25^th^ percentile are displayed in Table 2.

**Table 2:** General characteristics of the participants according to resilience level

	Resilience score above 75^th^ percentile (*n* = 60)	Resilience score between 75^th^ and 25^th^ percentile (*n* = 90)	Resilience score below 25^th^ percentile (*n* = 57)	*p* value
Age, (years)	36.0 [5.0]	37.0 [4.0]	36.0 [3.0]	n.s.
Body mass index, kg/m^2^	24.50 [6.33]	23.43 [15.06]	23.87 [15.98]	n.s.
Waist circumference, cm	81.5 [11.0]	80.0 [17.0]	78.0 [11.3]	n.s.
Smoking habit	25.0%	16.7%	15.8%	n.s.
Years attempting to get pregnant	3.11 (1.64)	3.28 (1.91)	3.37 (2.19)	n.s.
Previous IVF attempts	58.33%	48.89%	42.11%	n.s.
History				
Previous miscarriage	28.8%	21.1%	22.8%	n.s.
Previous live birth	3.4%			
Nulligravida	67.8%	78.9%	77.2%	n.s.
Cause of infertility				n.s.
Female	15.0%	21.1%	21.1%	
Male	28.3%	38.9%	35.1%	
Multiplefactors	26.7%	26.7%	22.8%	
Unexplained	30.0%	13.3%	21.1%	

Data are presented as %, mean (standard deviation) and median [interquartile range]. Chi square a *T* test were employed

Perceived stress showed a positive correlation with number of IVF attempts (*r* = 1.77; *p* = 0.012) and depressed mood (*r* = 0.717; p < 0.001). Lastly, depressed mood showed a positive correlation with sleep disturbance (*r* = 0.611; *p* < 0.001).

**Fig. 1:**
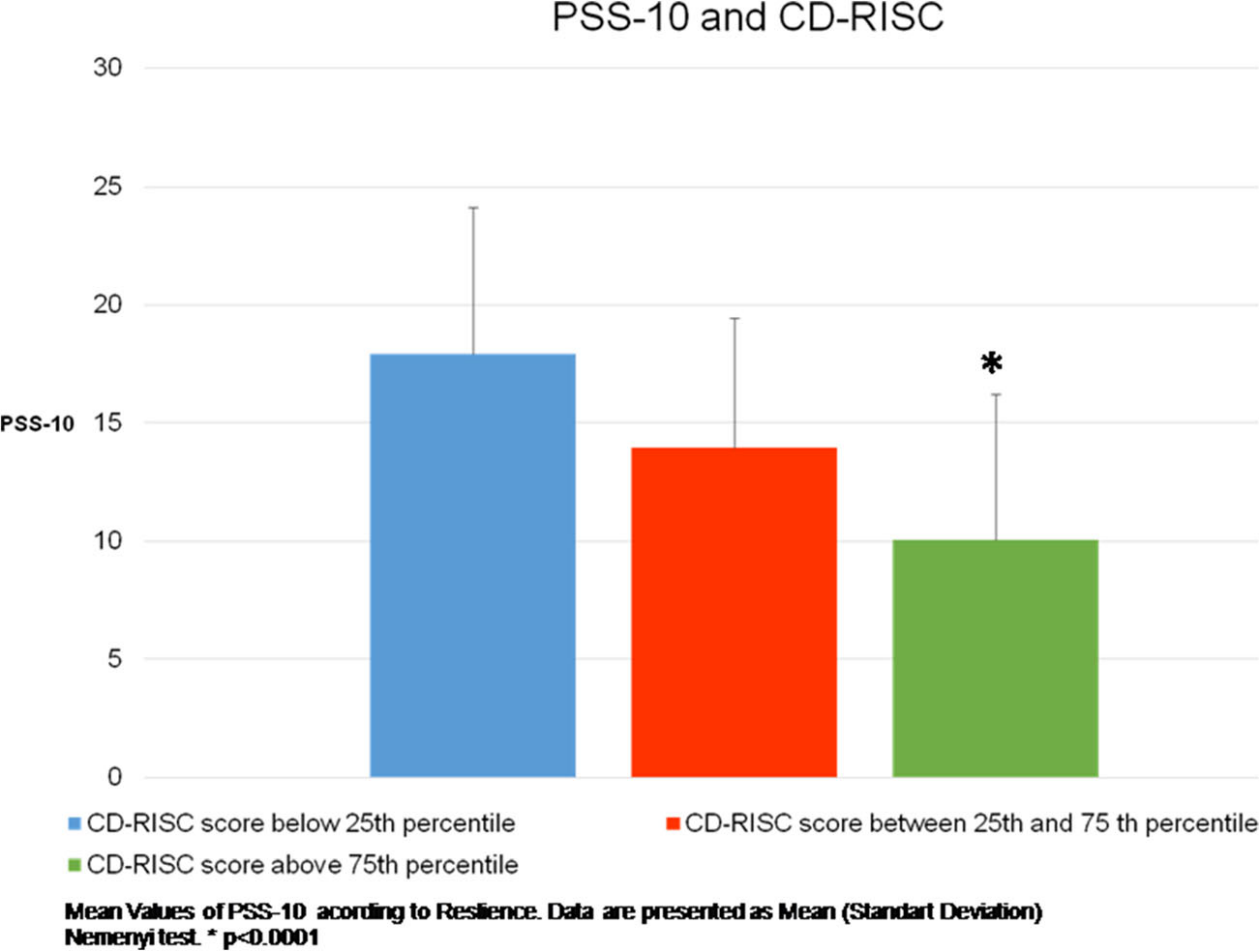
Mean PSS-10 scores and CD-RISC scores ranked according percentiles

For the total sample, the relationship between CD-RISC scores ranked according percentiles and mean PSS-10 scores revealed that women with strong resilience had lower perceived stress (Fig. 1). When the sample was assessed according the number of cycles, the mean scores for the CD-RISC, CESD-10, and JSS did not change between the first and subsequent IVF attempts, but the mean PSS-10 score increased after obtaining negative results (Table 3).

**Table 3:** Changes in perceived stress, depressed mood and sleep disturbance according IVF attempt

	1^st^ cycle(*n* = 105)	2^nd^ cycle or more (*n* = 103)	*p* value
Resilience score	28.04 (4.90)	28.93 (5.05)	ns
PSS-10	12.95 (6.36)	15.16 (6.64)	0.021
CESD-10	8.8%	17.3%	n.s.
JSS	7.7%	14.3%	n.s.

Data are presented as %, mean (standard deviation)

**Fig. 2:**
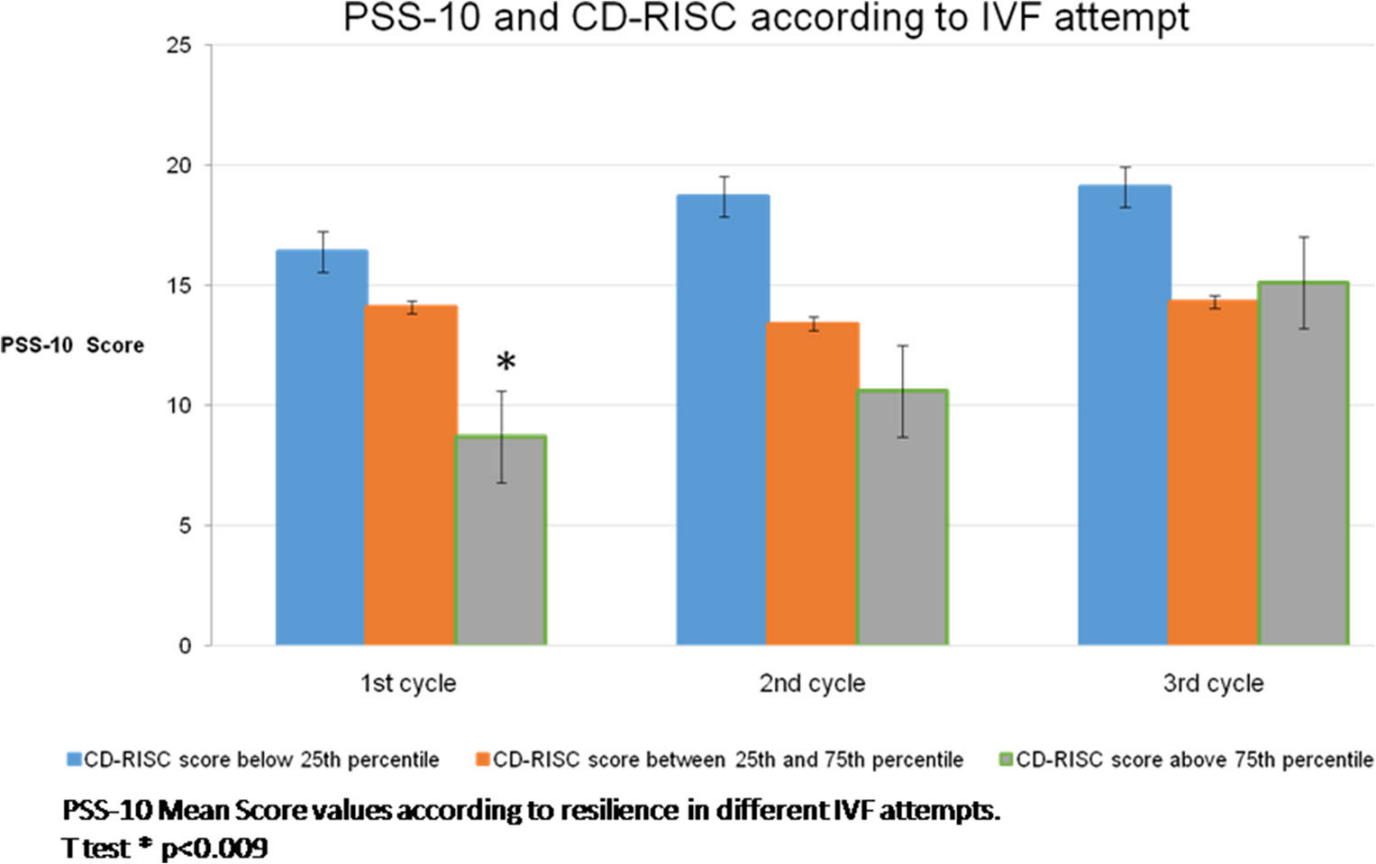
PSS and CD-RISC variations according to IVF attempt

**Fig. 3:**
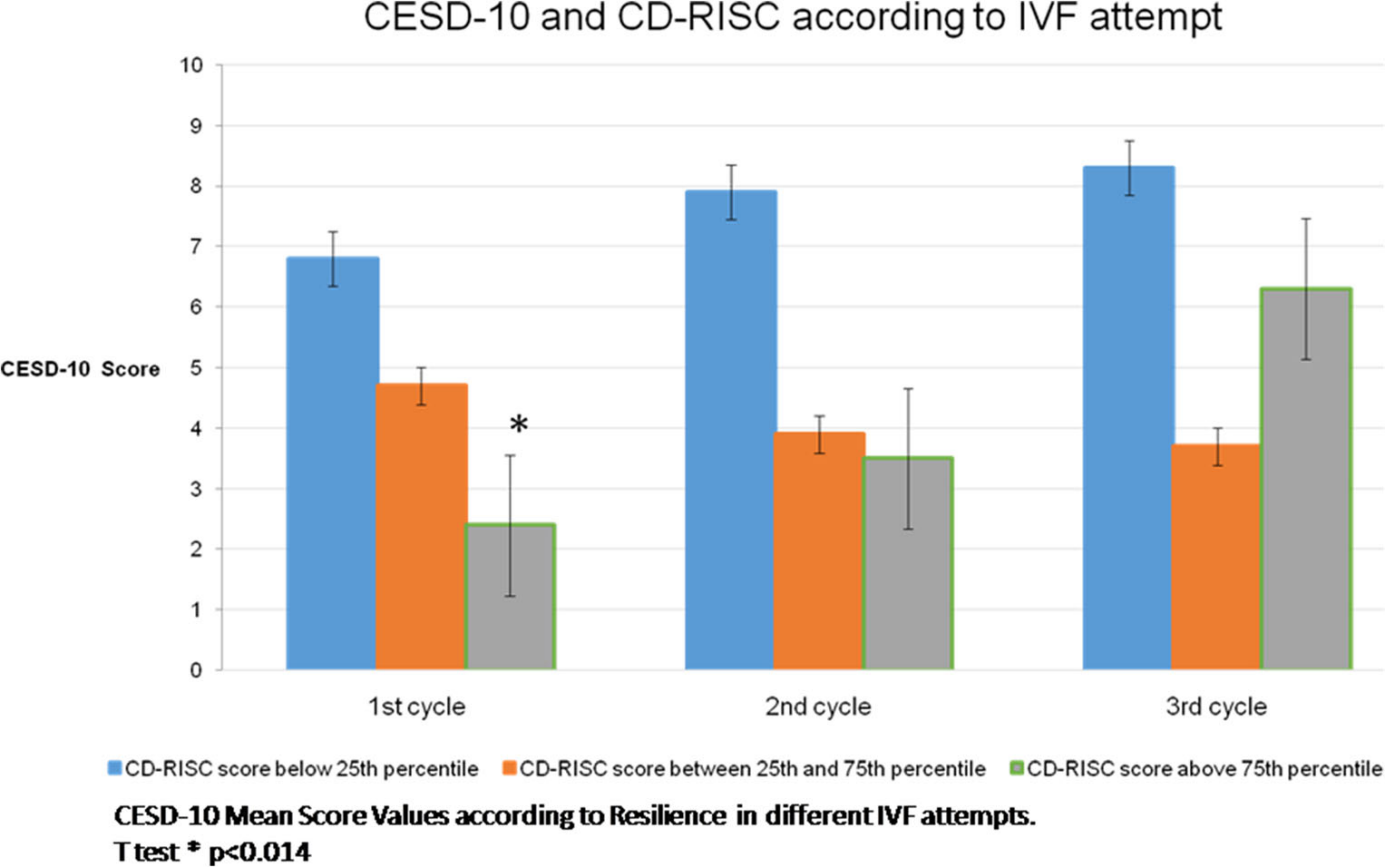
CESD-10 and CD-RISC variations according to IVF attempt

After splitting the sample according to CD-RISC percentiles, differences between the mean scores of PSS-10 and CSD-10 score mean were observed only at the first IVF attempt and the observed protective effect of high resilience scores disappeared following a negative IVF outcome (Figs. 2 and 3).

## Discussion

When a couple fails to conceive spontaneously, both partners experience sadness and disappointment. Infertility can disrupt a woman’s life goals, and those who experience infertility and the need to undergo IVF treatment are often anxious and depressed because of their infertility and the uncertainties of the treatment with which they are faced [18]. Although some studies have found no association between emotional stress and IVF results [19, 20], others report that the outcome of IVF treatment may be dependent on psychological stress [21, 22]. However, since resilient individuals feel confident that they can overcome their emotions, highly resilient women receiving fertility treatment could experience less psychological stress during IVF treatments and more rapid recovery over time following a failed attempt [23, 9].

In the present research, perceived stress was positively correlated with the number of IVF attempts, and women with strong resilience showed lower levels of perceived stress at the first IVF attempt, supporting the potential protective effect of resilience on stress and depressed mood. These data are in accord with the findings of a recent study reporting the outcomes associated with resilience trajectories during the first IVF/ICSI treatment cycle [24].

Failure of IVF treatment may result in short-term symptoms of depression and a number of research studies have found an association between high stress levels during fertility treatments and short-term symptoms of depression and a high rate of withdrawal from IVF treatments [25, 26]. In the present study, we also observed a positive correlation between perceived stress and depressed mood. In contrast, resilience, marital quality, and other psychosocial variables could facilitate recovery after a negative IVF outcome [23, 7, 27] while it has also been suggested that resilience declines over time, particularly for repeat IVF patients [28]. Our data partially support those findings, since a pilot study found that the putative protective effect of resilience on perceived stress or depressed mood was also lost after a negative IVF outcome.

Our study has several limitations. First, we employed a cross-sectional study design and the identified associations might be difficult to interpret, particularly given that resilience did not continue to be a protective factor after one failed cycle of IVF, meaning that other variables might have been responsible for the low scores. Second, we did evaluate any biomarkers of stress, although it should be noted that rigorous research has found no support for a relationship between measures of psychological distress and stress hormones in fertility patients. Lastly, our sample was limited to women who received publicly funded IVF treatment after a long period of time spent on a waiting list and who had only three IVF attempts.

Despite those limitations, our findings are particularly interesting if it is considered that women’s distress regarding fertility begins long before IVF, and many women already present signs of depressed mood at the time of beginning IVF. Theoretically, an evaluation of resilience prior to starting infertility treatment could help to provide emotional support to women with low resilience during IVF and to reduce the emotional impact of this procedure.

## Conclusion

The findings of the present study have demonstrated that while women with high resilience are less likely to suffer from perceived stress or depressed mood during their first IVF attempt, this protective effect appears to be lost after a negative outcome. Overall, these findings indicate that while resilience could initially be beneficial, it is ineffective following an unsuccessful IVF attempt and women might need additional support to manage their recovery. Moreover, resilience assessment prior to starting infertility treatment could help to provide emotional support to women with low resilience during IVF procedures and reduce the emotional impact and withdrawal rate. Nonetheless, further longitudinal research studies are needed to confirm these observed relationships.

## Data Availability

Raw data on Excel format can be availed on reasonable request from the corresponding author.

## References

[1] Infertility Workup for the Women’s Health Specialist: ACOG Committee Opinion, Number 781. Obstet Gynecol. 2019;133(6):e377–84. 10.1097/AOG.0000000000003271 31135764

[2] Definitions of infertility and recurrent pregnancy loss: a committee opinion. Fertil Steril. 2020;113(3):533–5. 10.1016/j.fertnstert.2019.11.025 32115183

[3] Vander Borght M, Wyns C. Fertility and infertility: definition and epidemiology. Clin Biochem. Elsevier Inc. 2018;62:2–10. 10.1016/j.clinbiochem.2018.03.012 29555319

[4] Hart RJ (2016). Physiological aspects of female fertility: role of the environment, modern lifestyle, and genetics. Physiol Rev.

[5] Aimagambetova G, Issanov A, Terzic S, Bapayeva G, Ukybassova T, Baikoshkarova S, et al. The effect of psychological distress on IVF outcomes: reality or speculations? PLoS One. 2020;15(12):e0242024. 10.1371/journal.pone.0242024. 10.1371/journal.pone.0242024 PMC7735622 33315878

[6] Massarotti C, Gentile G, Ferreccio C, Scaruffi P, Remorgida V, Anserini P (2019). Impact of infertility and infertility treatments on quality of life and levels of anxiety and depression in women undergoing in vitro fertilization. Gynecol Endocrinol.

[7] Gameiro S, Van Den Belt-Dusebout AW, Smeenk JMJ, Braat DDM, Van Leeuwen FE, Verhaak CM (2016). Women’s adjustment trajectories during IVF and impact on mental health 11-17 years later. Hum Reprod.

[8] Gabnai-Nagy E, Papp G, Nagy BE (2020). The influence of emotional state and coping ability on the outcome of the assisted reproductive technology among infertile couples. Psychiatr Hung.

[9] Vatanparast M, Yasini Ardekani SM, Anvari M, Kalantari A, Yaghmaie F, Royani Z. Resilience as the predictor of quality of life in the infertile couples as the most neglected and silent minorities. J Reprod Infant Psychol. 2020:1–12. 10.1080/02646838.2020.1843613 33167710

[10] Connor KM, Davidson JRT (2003). Development of a new resilience scale: the Connor-Davidson Resilience scale (CD-RISC). Depress Anxiety.

[11] Notario-Pacheco B, Solera-Martínez M, Serrano-Parra MD, Bartolomé-Gutiérrez R, García-Campayo J, Martínez-Vizcaíno V (2011). Reliability and validity of the Spanish version of the 10-item Connor-Davidson Resilience Scale (10-item CD-RISC) in young adults. Health Qual Life Outcomes.

[12] Cohen S. Perceived stress scale [Internet]. [cited 2020 Jun 28]. Available from: https://biadmin.cibersam.es/Intranet/Ficheros/GetFichero.aspx?FileName=466_0fcf150b-b08b-46a1-9f69-dc287bec1d54.pdf. Accessed 13 Sept 2021.

[13] Remor E (2006). Psychometric properties of a European Spanish version of the Perceived Stress Scale (PSS). Span J Psychol.

[14] Christensen H, Batterham PJ, Grant J, Griffiths KM, MacKinnon AJ. A population study comparing screening performance of prototypes for depression and anxiety with standard scales. BMC Med Res Methodol. 2011;11. 10.1186/1471-2288-11-154 PMC3235985 22103584

[15] Llaneza P, García-Portilla MP, Llaneza-Suárez D, Armott B, Pérez-López FR (2012). Depressive disorders and the menopause transition. Maturitas..

[16] Lallukka T, Dregan A, Armstrong D (2011). Comparison of a sleep item from the general health questionnaire-12 with the Jenkins Sleep Questionnaire as measures of sleep disturbance. J Epidemiol.

[17] Ornat L, Martínez-Dearth R, Chedraui P, Pérez-López FR (2014). Assessment of subjective sleep disturbance and related factors during female mid-life with the Jenkins Sleep Scale. Maturitas..

[18] Hodis HN, Mack WJ, Azen SP, Lobo RA, Shoupe D, Mahrer PR, Faxon DP, Cashin-Hemphill L, Sanmarco ME, French WJ, Shook TL, Gaarder TD, Mehra AO, Rabbani R, Sevanian A, Shil AB, Torres M, Vogelbach KH, Selzer RH (2003). Hormone therapy and the progression of coronary-artery atherosclerosis in postmenopausal women. N Engl J Med.

[19] Maroufizadeh S, Navid B, Omani-Samani R, Amini P (2019). The effects of depression, anxiety and stress symptoms on the clinical pregnancy rate in women undergoing IVF treatment. BMC Res Notes.

[20] Miller N, Herzberger EH, Pasternak Y, Klement AH, Shavit T, Yaniv RT, Ghetler Y, Neumark E, Eisenberg MM, Berkovitz A, Shulman A, Wiser A (2019). Does stress affect IVF outcomes? A prospective study of physiological and psychological stress in women undergoing IVF. Reprod BioMed Online.

[21] Cesta CE, Viktorin A, Olsson H, Johansson V, Sjölander A, Bergh C (2016). Depression, anxiety, and antidepressant treatment in women: association with in vitro fertilization outcome. Fertil Steril.

[22] Bapayeva G, Aimagambetova G, Issanov A, Terzic S, Ukybassova T, Aldiyarova A, Utepova G, Daribay Z, Bekbossinova G, Balykov A, Laganà AS, Terzic M (2021). The effect of stress, anxiety and depression on in vitro fertilization outcome in kazakhstani public clinical setting: a cross-sectional study. J Clin Med.

[23] Chochovski J, Moss SA, Charman DP (2013). Recovery after unsuccessful in vitro fertilization: the complex role of resilience and marital relationships. J Psychosom Obstet Gynecol.

[24] Li G, Jiang Z, Kang X, Ma L, Han X, Fang M. Trajectories and predictors of anxiety and depression amongst infertile women during their first IVF/ICSI treatment cycle. J Psychosom Res. 2021;142. 10.1016/j.jpsychores.2021.110357. 10.1016/j.jpsychores.2021.110357 33508704

[25] Domar AD. Impact of psychological factors on dropout rates in insured infertility patients. Fertil Steril. Elsevier Inc. 2004;81:271–3. 10.1016/j.fertnstert.2003.08.013 14967355

[26] Olivius C, Friden B, Borg G, Bergh C (2004). Why do couples discontinue in vitro fertilization treatment? A cohort study. Fertil Steril.

[27] Kang X, Fang M, Li G, Huang Y, Li Y, Li P (2021). Family resilience is a protective buffer in the relationship between infertility-related stress and psychological distress among females preparing for their first in vitro fertilization–embryo transfer. Psychol Health Med.

[28] Turner K, Reynolds-May MF, Zitek EM, Tisdale RL, Carlisle AB, Westphal LM (2013). Stress and anxiety scores in first and repeat IVF cycles: a pilot study. PLoS One.

